# Distribution and Oxidation Rates of Ammonia-Oxidizing Archaea Influenced by the Coastal Upwelling off Eastern Hainan Island

**DOI:** 10.3390/microorganisms10050952

**Published:** 2022-04-30

**Authors:** Hao Liu, Peng Zhou, Shunyan Cheung, Yanhong Lu, Hongbin Liu, Hongmei Jing

**Affiliations:** 1 CAS Key Laboratory for Experimental Study under Deep-Sea Extreme Conditions, Institute of Deep-Sea Science and Engineering, Chinese Academy of Sciences, Sanya 572000, China; liuh@idsse.ac.cn (H.L.); pengzjerry@163.com (P.Z.); 2 Department of Ocean Science, Hong Kong University of Science and Technology, Hong Kong, China; sycheungab@connect.ust.hk (S.C.); ylubf@connect.ust.hk (Y.L.); liuhb@ust.hk (H.L.); 3 HKUST-CAS Sanya Joint Laboratory of Marine Science Research, Chinese Academy of Sciences, Sanya 572000, China; 4 Southern Marine Science and Engineering Guangdong Laboratory, Zhuhai 519000, China

**Keywords:** ammonia oxidizing archaea, coastal upwelling, ammonia oxidation rate, *amoA* gene, distribution

## Abstract

Coastal upwelling causes variations in temperature, salinity and inorganic nutrients in the water column, consequently leading to the shift of microbial populations and their metabolic activities. Impacts of the eastern Hainan upwelling (EHU) on the ammonia-oxidizing archaea (AOA) were investigated based on the *amoA* gene using pyrosequencing and quantitative PCR at both DNA and cDNA levels, together with the determination of the ammonia oxidation (AO) rate measured with ^15^N-labelled ammonium. By comparing stations with and without upwelling influence, we found that coastal upwelling correlated with an increase in *amoA* gene abundance, the dominance of distinct clades for AOA communities at the respective gene and transcript levels, and a large increase in the proportion of the SCM1-like (*Nitrosopumilus maritimus*-like) cluster as well. The AO rates were generally higher in the deeper water (~25 m), which was in significant positive correlation with the proportion of cluster Water Column A (WCA) at the transcript level, indicating the potential contribution of this cluster to in situ ammonia oxidization. Our study demonstrated that coastal upwelling had a significant impact on the AOA community and ammonia oxidization rate; therefore, this physical forcing should be considered in the future assessment of the global nitrogen budgets and biogeochemical nitrogen cycles.

## 1. Introduction

Nitrification is a microbe-mediated process that sequentially oxidizes ammonia into nitrate and connects the biological nitrogen fixation and nitrogen loss processes in the marine nitrogen cycle. Ammonia oxidation (AO), the first and rate-limiting step of nitrification, is carried out by ammonia-oxidizing microbes. Ammonia-oxidizing archaea (AOA), which have a higher affinity to ammonia [1] and population abundance [2] than ammonia-oxidizing bacteria, act as the major ammonia oxidizers in various marine environments [3,4,5].

Since the first phylogenetic analysis of the AOA *amoA* gene in the ocean [6], water column A (WCA) [6] and water column B (WCB) [6] were reported as two major groups in oceanic waters, dominating in upper (<200 m) and deep (>200 m) layers, respectively [7,8]. Sublineages of WCA and WCB have a global distribution with varied distributional patterns and environmental determinants [9]. SCM1-like AOA (affiliated to the first isolated AOA-*Nitrosopumilus maritimus* SCM1) [10] was also frequently detected in high-latitude waters and euphotic zones [9]. The community composition and gene abundance of AOA have been reported to be strongly influenced by the associated hydrographic conditions such as water depth, ammonium concentration, light, temperature, and oxygen levels [11,12] in addition to being geographically varied [13,14]. In addition, the discrepancy of AOA diversity based on the *amoA* gene between the gene and gene transcript levels were reported [15,16]. A previous study retrieved a distinct AOA community at the RNA level, with a phylotype affiliated to *Nitrosomarinus* showing widespread expression in the coastal region of the Baltic Sea [17]. This highlights the necessity of conducting microbial community structure at both the gene and gene transcript levels, because the latter reflects metabolic active assemblages.

The ammonia oxidation process has been reported to be closely associated with the gene abundance of AOA, and mainly limited by ammonia concentration in seawater [7]. The AO rate was low in the surface and reached its maximum in the region of below 1% photosynthetically active radiation, and then decreased until becoming undetectable in water below 300 m in the Eastern Tropical North Pacific [18]. As a physical ocean process, upwelling brings cold and nutrient-rich deep water to the euphotic layer, and leads to changes in the microbial community composition and metabolic function in seawater [15,16]. Therefore, it would be reasonable to predict a shift in the community composition and gene abundance of AOA in response to the gradients of physicochemical properties in upwelling influenced coastal waters.

The eastern Hainan, upwelling is one of the strongest upwellings off the Hainan Island in the northern South China Sea [19]. The EHU is mainly caused by the Asian summer monsoon and generally occurs from April to September with a peak period from June to July [20,21]. We collected water samples from upwelling- and non-upwelling regions during the summer monsoon, and conducted a comparative study based on the *amoA* gene at both the DNA and cDNA levels. The ammonia oxidation rate using ^15^N tracing method was applied as well to elucidate the impact of upwelling on the phylogenetic diversity, gene abundance, and activities of AOA.

## 2. Materials and Methods

### 2.1. Sample Collection and Environmental Factor Measurement

**Figure 1:**
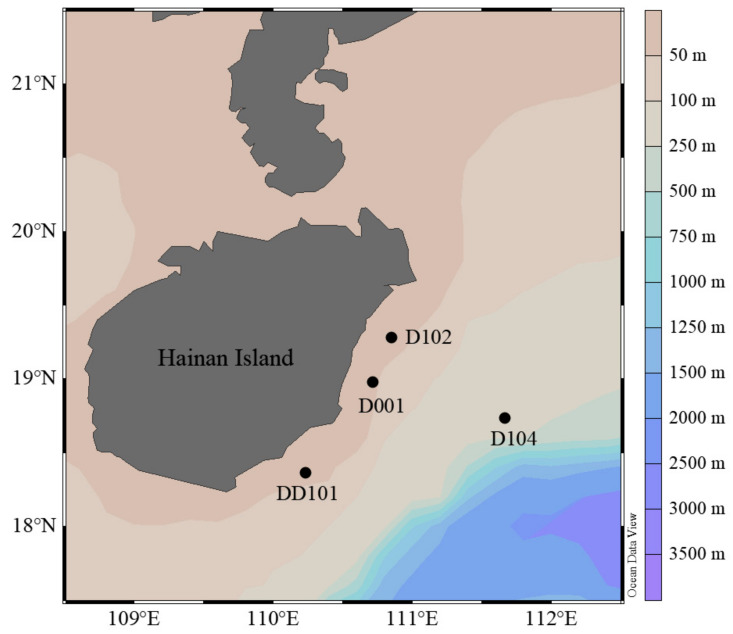
Location of the sampling stations in eastern Hainan Island during summer.

Seawater samples were collected from four stations in eastern Hainan during a cruise from 29 July to 7 August in 2015 (Figure 1). Stns. D001, D102, and DD101 were located in the upwelling region, while Stn. D104 was located in the non-upwelling region. Waters were collected from both 5 m and 25 m at Stns. D001 and DD101, and from 25 m at Stns. D102 and D104 using a CTD carousel water sampler with Niskin bottles (General Oceanics, Miami, FL, USA) and filtered through 0.22 μm pore-size polycarbonate membrane (47 mm, EMD Millipore, Billerica, MA, USA). The filters for RNA analysis were immersed in RNAlater solution (Ambion, Thermo Fisher Scientific, Corp., Waltham, MA, USA) immediately after filtration. All filters were then flash frozen and stored at −80 °C until extraction on land. Environmental parameters (temperature, salinity, depth) were recorded in situ with conductivity–temperature–depth (CTD, Sea-Bird Electronics, WA, USA). Concentrations of inorganic nutrients (such as nitrate, nitrite, ammonium, phosphate, and silicate) were measured with an auto-analyzer (QuAAtro, BLTEC. Co. Ltd., Osaka, Japan) which was calibrated with certified seawater nutrient reference material (RM; KANSO, Osaka, Japan).

### 2.2. DNA and RNA Extraction and cDNA Synthesis

Genomic DNA was extracted from the 0.22 µm polycarbonate filters with a PureLink Genomic DNA Mini Kit (Invitrogen, Thermo Fisher Scientific, Corp., Carlsbad, CA, USA), eluted into 100 µL Tris-EDTA (TE) buffer and stored at −80 °C. Total RNA was extracted from the 0.22 µm polycarbonate filters with the TRIzol plus RNA purification kit (Invitrogen). RNAlater was removed before the preparation with TRIzol Reagent, and the extracted RNA was eluted in 50 µL of elution buffer. The concentrations of DNA and RNA were measured with a NanoDrop 2000 Spectrophotometer (Thermo Scientific, Thermo Fisher Scientific, Corp., Waltham, MA, USA).

Before cDNA synthesis, purified total RNA was treated with DNase I (Invitrogen) and incubated at room temperature for 15 min to eliminate any potential DNA contamination. DNase I was then inactivated by heating at 65 °C for 10 min with 1 μL EDTA. Total RNA (up to 200 ng) was then reverse transcribed to cDNA using the SuperScript III first-strand cDNA synthesis kit (Invitrogen). A parallel reaction without SuperScript III RT was used as an RT-PCR negative control. Synthesized cDNA was further digested with 1 μL RNase H at 37 °C for 20 min to remove residual RNA, and it was then used for subsequent PCR amplification. Non-RT samples were used as negative controls.

### 2.3. Quantitative PCR

The abundance of the *amoA* gene and gene transcripts was determined by the StepOnePlus quantitative PCR (qPCR) system (Applied Biosystems, Inc., Carlsbad, CA, United States) with 25 µL of the SYBR^®^ Premix Ex Taq TM kit (Takara Bio, Inc., Shiga, Japan), 0.3 µM of the *amoA*196F (5′-GGWGTKCCRGGRACWGCMAC-3′) and *amoA*277R (5′-CRATGAAGTCRTAHGGRTADCC-3′) primers [22], and 2 µL of each DNA/cDNA as the template. The standard curve for absolute quantification was constructed using plasmid amplicons that were quantified on an Agilent 2100 bioanalyzer using DNA 7500 chips, according to the manufacturer’s protocol (Agilent Technologies, Inc., Santa Clara, CA, USA). Triplicate qPCR reactions were performed for each sample with efficiencies of approximately 102.16%, and the gene copy number was normalized to the quantity of the gene and gene transcripts. The theoretical copy number was calculated to the size of the input PCR amplicon. In parallel, negative controls without reverse transcriptase and template were also prepared for the qPCR reactions, and no amplicons were produced. The gene copy number was normalized to the quantity of the gene against a standard curve, which was constructed with a series concentration of quantified linear plasmid. The quantity of serial diluted plasmids in the standard curve was determined with NanoDrop 2000C spectrophotometer (Thermo Scientific, Wilmington, DE, USA)

### 2.4. 454 Pyrosequencing and Bioinformatics Analysis

For each genomic DNA and cDNA sample, independent triplicates were extracted as templates to amplify the *amoA* gene using Arch-*amoA*F (5′-STAATGGTCTGGCTTAGACG-3′) and Arch-*amoA*R (5′-GCGGCCATCCATCTGTATGT-3′) primers following the PCR previously described protocols [6]. Barcodes were incorporated between the adapter and forward primer. Nuclease-free water was used as the negative control in each reaction. Triplicate PCRs were performed for each sample, and the amplicons were pooled and subsequently purified with the illustra GFX^TM^ PCR DNA and Gel Band Purification kit (GE Healthcare, Little Chalfont, Bucks, United Kingdom). An amplicon library was constructed with equimolar concentrations of the amplicons and emPCR was conducted according to the Rapid Library preparation kit instructions (Roche, Basel, Switzerland). DNA beads were successfully deposited onto the PicoTiterPlate and sequenced with a GS Junior system (Roche).

The *amoA* sequences generated in this study were processed using the microbial ecology community software program mothur [23]. Sequences were de-noised, and the barcode and forward primer sequences were removed simultaneously with the shhh.seqs (sigma value = 0.01) and trim.seqs scripts, and chimeric sequences were identified with chimera.uchime [23]. Reads shorter than 300 bp in length and sequences containing undetermined nucleotides were removed. The phylogenetic distances between these high-quality sequences were calculated with mothur [23], and operational taxonomic units (OTUs) were generated with 97% DNA sequence similarity as the cutoff value. OTUs that contained just one sequence were removed. Diversity index (Shannon–Wiener index, Simpson index, and Peilou’s evenness) and Coverage were calculated with the “vegan” package in R [24]. The top OTUs were selected based on relative abundance ≥ 0.1% [25] for subsequent analysis, and the remaining OTUs were treated as a minority group. In total, 293 OTUs were generated. Additionally, the top 43 OTUs with relative abundance > 0.1% were classified as abundant OTUs. The top 43 OTUs accounted for 93.2% of the total sequences and the rest of the OTUs were classified as “rare” OTUs. Therefore, the “rare” in the figure of community structure of AOA at the DNA and cDNA levels is the agglomerated relative abundance of “rare” OTUs.

The representative sequences of the top OTUs, the selected reference sequences [17,26], and the environmental sequences of the *amoA* gene from the NCBI database were used to construct a maximum-likelihood (ML) tree using the MEGA 7.0 (molecular evolutionary genetics analysis) software [27]. The DNA sequences were codon-aligned and a model test was conducted to select the best fit DNA substitution model for the construction of the ML tree. Based on the Bayesian Information Criterion calculation, the Tamura 3-parameter model, using discrete Gamma distribution with the assumption that a certain portion of sites are evolutionarily invariable (T92+G+I), was selected.

### 2.5. Nitrification Rate Measurements

Water samples for the AO rate measurements were collected from two to three depths in the upper 75 m (shown in the table of the vertical ambient nutrients concentrations and ammonia-oxidizing rates in upwelling and non-upwelling regions in the eastern Hainan Island during summer) and incubated following the protocol of Ward et al. [28]. Nitrification was measured by incubating ^15^NH_4_^+^ amended seawater in duplicate with 200 mL high-density polyethylene (HDPE) bottles in the dark for 24 h; and the temperature was controlled by running seawater. For each HDPE bottle, ^15^NH_4_Cl was injected to reach a final concentration of 20 nmol/L. After tracer addition and homogeneous mixing, ~50 mL water was immediately taken out from the incubation bottle and then filtered through a 0.22 μm disposable syringe filter (Sterivex, Millipore). The filtrates were frozen to serve as t0 samples. The other filtrates were collected after incubation as termination (t) samples and stored at −20 °C for downstream ^15^NO_x_^−^ (^15^NO_3_^−^+ ^15^NO_2_^−^) analysis [29]. The nitrification rates were calculated using the following equation:

(1) $$\mathrm{AO}\;=\frac{\left(R_{t}{\mathrm{NO}}_{x}{}^{-}\times \left[{\mathrm{NO}}_{x}{}^{-}\;\right]t\right)-({\mathrm{NO}}_{x}{}^{-}\times \left[{\mathrm{NO}}_{x}{}^{-}\right]t_{0}}{t-t_{0}}\times \frac{\left(14_{{\mathrm{NH}}_{4}{}^{+}}\right)+\left(15_{{\mathrm{NH}}_{4}{}^{+}}\right)}{15_{{\mathrm{NH}}_{4}{}^{+}}}$$

In Equation (1), AO is the nitrification rate. R_t0_NO_x_^−^ and R_t_NO_x_^−^ are the ratios (%) of ^15^N in the NO_x_^−^ pool measured at the initial (*t_0_*) and termination (*t*) of the incubation. [NO_x_^−^]t_0_ and [NO_x_^−^]t are the concentrations of NO_x_^−^ at the outset and termination of the incubation, respectively. (^14^NH_4_^+^) is the ambient NH_4_^+^ concentration. (^15^NH_4_^+^) is the final ammonium concentration after the addition of the stable isotope tracer (^15^NH_4_^+^). The NO_x_^−^ was completely converted to N_2_O by a single strain of denitrifying bacteria (*Pseudomonas aureofaciens*; ATCC no.13985) which lacks N_2_O reductase activity [29]. The converted N_2_O was further analyzed using an isotope ratio mass spectrometer (IRMS; Thermo Fisher Scientific DELTA V Plus) to calculate the isotopic composition of NO_x_^−^ [29,30,31].

### 2.6. Statistical Analysis and Accession Number

Non-metric multidimensional scaling (nMDS) analysis was performed using the “vegan” and visualized via “ggplot2” packages in R [24,32]. Then, ANOSIM was performed to test the significance of community differences among multiple groups. In addition, Spearman correlation coefficients between the environmental variables and the proportions of different clusters were calculated using the Graphpad Prism software package version 8.0 (GraphPad Software Inc., San Diego, CA, USA) after the data were square-rooted transformed. Values of *p* < 0.05 and *p* < 0.01 were considered to indicate different levels of statistical significance. All the *amoA* sequences obtained from this study were deposited in the National Center for Biotechnology Information (NCBI) Sequence Read Archive (SRA) under the accession number PRJNA732652. 

## 3. Results

### 3.1. Hydrographic Conditions of Sampling Stations

**Figure 2:**
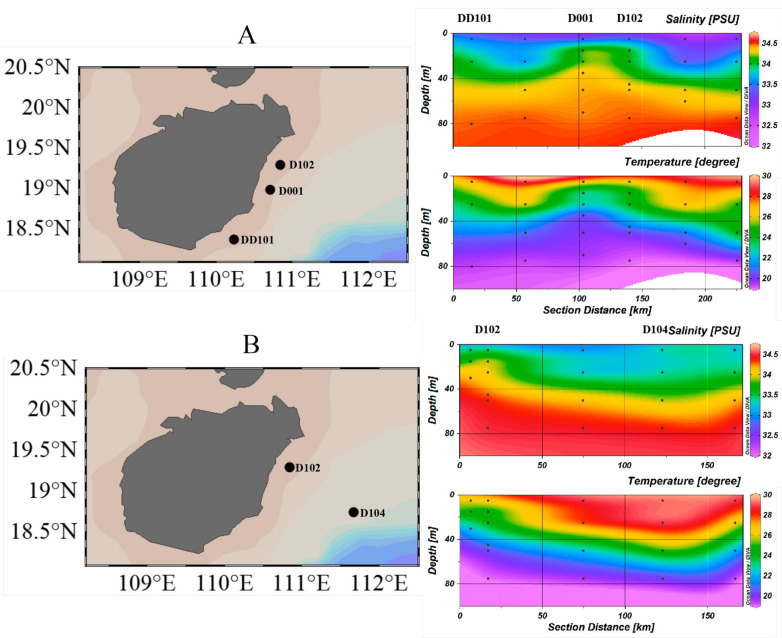
Temperature and salinity parameters at the sampling stations in eastern Hainan Island during summer: (**A**) salinity and temperature data for Stns. DD101, D001, and D102 represent the upwelling zone; and (**B**) salinity and temperature data of Stn. D104 represent the non-upwelling zone.

Compared with the surrounding waters, lower water temperature and higher salinity were detected at Stns. D001, D102 and DD101 (Figure 2A), while temperature and salinity at Stn. D104 were almost the same as the surrounding waters (Figure 2B). Concentrations of ambient inorganic nutrients, such as NH_4_^+^, NO_2_^−^, NO_x_^−^ and SiO_3_^2^^−^, at the same depths of the Stn. D102 were significantly higher than those at Stn. D104. Stns. D001, D102, and DD101 exhibited significantly lower temperature as well as higher salinity and inorganic nutrient concentrations, indicating an impact of the EHU, while Stn. D104 seemed unchanged by the EHU (Table S1).

### 3.2. Diversity of AOA

**Figure 3:**
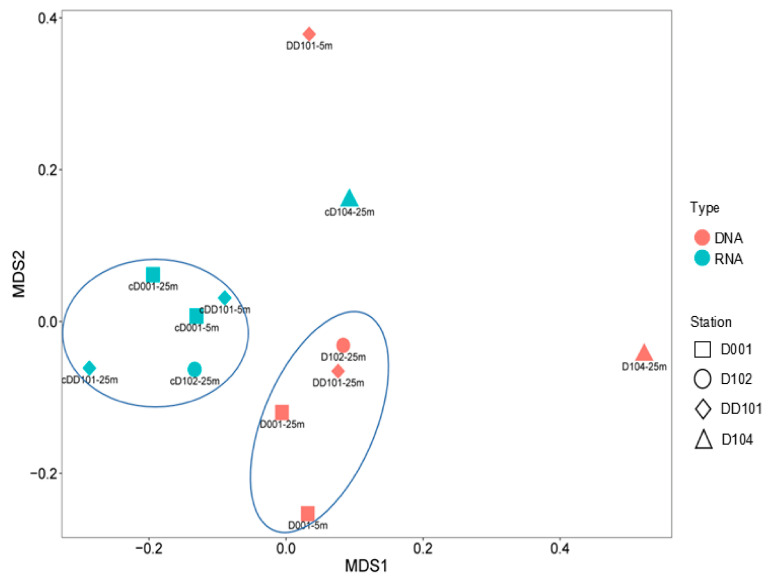
Grouping of different AOA communities according to Bray–Curtis dissimilarity using non-linear multidimensional scaling (nMDS).

Sequences generated were normalized to 1200 quality reads for each sample (Table 1). In terms of the community diversity, higher was shown at 25 m than at 5 m, and higher was found at the non-upwelling station than the upwelling stations. There was no significant difference in terms of community diversity between the DNA and cDNA levels, except for Stn. D104 at 25 m, which showed the highest number of OTUs (138), *J* (Peilou’s evenness, 0.83), Shannon (4.09), and Simpson (0.97) diversity at the DNA level (Table 1). In addition, coverage for all samples exceeded 96%, indicating sufficient sampling efforts were applied in this study. Based on nMDS clustering analysis, all the DNA samples were clustered together except for Stns. DD101 at 5 m and D104 at 25 m, and all the cDNA samples were clustered together except for Stn. D104 at 25 m (Figure 3).

### 3.3. Phylogeny and Community Composition of AOA

**Figure 4:**
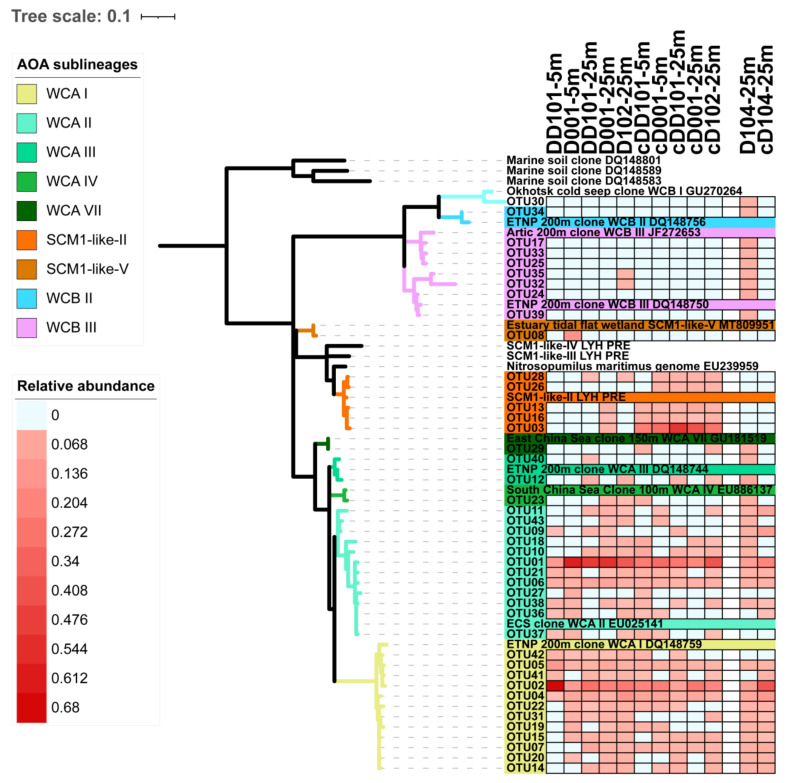
Phylogenetic maximum-likelihood tree of the AOA *amoA* gene sequences based on the top 43 OTUs obtained from samples collected in the eastern Hainan Island during summer. The associated heatmap was generated based on the relative abundance of top OTUs at the DNA and cDNA levels. Samples listed from left to right span from the upwelling region to the non-upwelling region.

In our study, a total of 735 OTUs were obtained and phylogenetically identified, and the community structure of AOA was obtained (Figure 4). Phylogenetic tree based on the top 43 OTUs demonstrated that the AOA sequences retrieved mainly fell into three clusters, i.e., the WCA (28 OTUs), WCB (9 OTUs), and SCM1-like (6 OTUs). Cluster WCA was predominant in all samples and was composed of five sublineages, WCA I (12 OTUs), WCA II (12 OTUs), WCA III (2 OTUs), WCA Ⅳ (OTU23), and WCA Ⅶ (OTU29). Cluster WCB consisted of three sublineages, namely WCB I (OTU30), WCB II (OTU34), and WCB III (7 OTUs). SCM1-like II (5 OTUs) and SCM1-like Ⅴ (OTU08) are the two sublineages of cluster SCM1-like (Figure 4).

**Figure 5:**
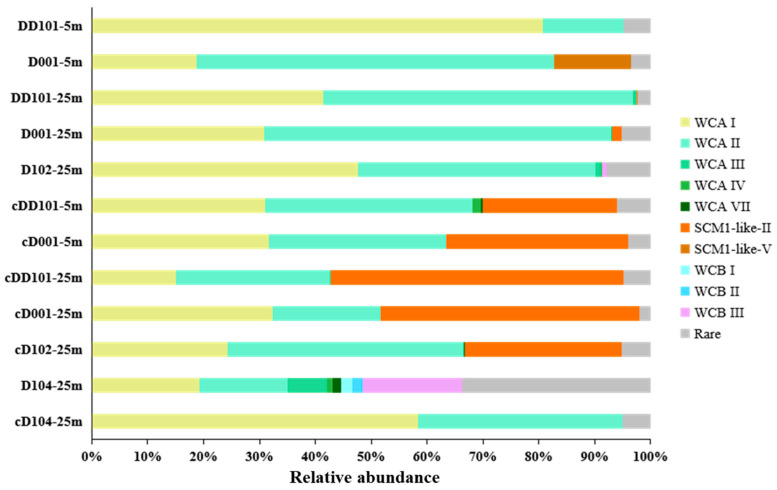
Community structure of AOA at the DNA and cDNA levels for samples collected from different stations in the eastern Hainan Island during summer.

At the DNA level, WCA I and WCA II dominated at all stations; WCB I only appeared at the Stn. D104 at 25 m; and WCB was mainly founded at Stn. D104 at 25 m (Figure 5). SCM1-like Ⅴ just appeared at Stn. D001 at 5 m, while SCM1-like II was found at the deeper water layer (25 m) of Stns. D001 and D102 (Figure 4). At the cDNA level, WCA I and WCA II predominated at all stations except for Stn. DD101 (25 m). Comparatively, at the upwelling stations, WCA I and WCA II had a higher relative abundance at the DNA level, while cluster SCM1-like had a higher relative abundance proportion at the cDNA level (Figure 5).

### 3.4. Abundance of AOA

**Figure 6:**
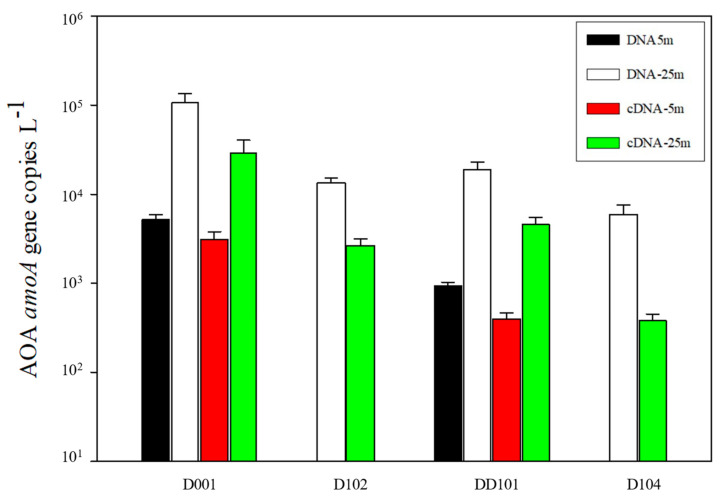
The abundance of the *amoA* gene quantified by qPCR at the DNA and cDNA levels in the eastern Hainan Island during summer. The error bars represent standard deviations.

The copy number of the *amoA* gene was in the range of 9.38 × 10^2^~1.07 × 10^5^ copies L^−1^ at the DNA level and was one magnitude (~10 times) higher than its corresponding cDNA level (Figure 6). Considering the abundance of *amoA* gene at both the DNA and cDNA levels as a whole, Stn. D001 was the highest among the four stations, and the shallow layer (5 m) was significantly lower (*p* < 0.01) than the deep layer (25 m) at Stns. D001 and DD101. The abundances of amoA gene in the upwelling zone was significantly higher (*p* < 0.05) than those in the non-upwelling zone at both the DNA and cDNA levels.

### 3.5. Ammonia Oxidation Rates

In general, the ambient NH_4_^+^ concentration at the upwelling stations was much higher than the non-upwelling station, especially obvious between Stn. 102 with Stn. 104 at the same depths (Table 2). AO was 1.53 ± 1.11 nmol L^−1^ d^−1^ at 5 m and 0.77–5.63 nmol L^−1^ d^−1^ at 25 m, reached 11.97 nmol L^−1^ d^−1^ at 50 m and peaked at 75 m with 52–112 nmol L^−1^ d^−1^ at the upwelling stations. Comparatively, the AO rate was low at 5 m (0.13 ± 0.01 nmol L^−1^ d^−1^) and increased at 25 m, and slightly decreased at 50 m at the non-upwelling Stn. D104. Remarkable differences in the NH_4_^+^ concentration and AO rates were found between upwelling Stn. D102 and non-upwelling Stn. D104. At the upwelling stations, AO rates at 5 m–75 m had linear relationships with the concentrations of NO_x_^−^, (AO)_rates_ = −2.1810 + 15.7048 × (NO_x_^−^)con (R = 0.9962, *p* < 0.01), which was not exhibited in the non-upwelling Stn. D104 (Table 2).

## 4. Discussion

### 4.1. Upwelling Effects on AOA Abundance

Gene abundance of *amoA* was one magnitude higher than their gene transcripts in our study, further proving the necessity of investigating microbial communities at both the DNA and cDNA levels. Since most studies of AOA abundance were conducted at the DNA level [33,34], discrepancies in the abundance and diversity of AOA between the DNA and cDNA levels have recently been revealed [26,35,36], and the cDNA level only reflects the active fraction of the microbial population [35,36]. The abundance of the *amoA* gene and gene transcript at 25 m was significantly higher than that at 5 m (*p* < 0.05), which might be caused by light inhibition [26,37] or competition with phytoplankton for NH_4_^+^ uptake [38]. Perhaps the same reasons caused the low abundance of the *amoA* gene and gene transcript of AOA in our study compared to previous studies [26,35,36]. AOA *amoA* gene abundance was always higher than their gene transcript at both the upwelling and non-upwelling stations, suggesting that only a fraction of AOA was metabolically active, as also found in a previous study [36]. The gene abundance of AOA was always higher at the upwelling station than that of the non-upwelling station, which might reflect the upwelling effect. Upwelling was reported to be able to significantly enhance the abundance of WCB in a seasonal oxygen-deficient coastal upwelling system of the eastern South Pacific Ocean [16]. This might be because nutrient-rich deep waters were taken to the shallow waters by upwelling and subsequently promote the growth of AOA.

### 4.2. Upwelling Effects on AOA Community Structure

Community diversity of AOA at 25 m was significantly higher than that of 5 m, possibly due to the light inhibition which occurred in the latter [37,39]. At the upwelling-stations, distinct clades of community for the respective DNA and cDNA levels formed as demonstrated by nMDS analysis, which was not observed at the non-upwelling station, suggesting an influence of upwelling on the AOA community.

Cluster WCA (I and III) was found to be predominant at all stations at the cDNA level, which was consistent with a previous study [26]. This was not surprising because all the cDNA samples were collected from shallow waters which would be more suitable for the shallow-water-adapted WCA than WCB [6]. At non-upwelling Stn. D104, cluster WCB ((I, II and III) was only detected at the DNA level but absent from the cDNA level, suggesting these sublineages had no transcriptional activity, although they accounted for approximately 50% proportion of the community.

SCM1-like cluster accounted for a higher proportion at the cDNA level at the upwelling stations, suggesting this cluster had strong transcriptional activity possibly induced by upwelling. Negative Spearman correlation (R = −0.6259, *p* < 0.05), found between the temperature and relative abundance of SCM1-like-Ⅱ, implied that this sublineage had a fast response to the environmental variations induced by upwelling. Cluster SCM1-like was reportedly well adapted to life under extreme nutrient limitation, sustaining high specific oxidation rates in open oceans [40]. High transcriptional activity for certain SCM1-like sublineages was found in Pearl River estuary [36]. However, transcript levels might not reflect the real ecological functions. The proportion of cluster SCM1-like at both the DNA and cDNA levels has no significant correlation with AO rates; the possible reasons behind this still need further exploration.

### 4.3. Ammonia Oxidation Rates during Coastal Upwelling

EHU upwelling was accompanied by higher NH_4_^+^ concentrations even at the nano molar level in our study. Concentrations of the inorganic NH_4_^+^ in the upwelling-influenced Stn. D102 were significantly higher (*p* < 0.01) than those of non-upwelling Stn. D104, and the upwelled deep water continuously provides substrate (NH_4_^+^) for AO processes. In the euphotic zone, nitrifiers and phytoplankton compete for ambient NH_4_^+^ [38]. If the NH_4_^+^ was directly oxidized by ammonia oxidizers, the NO_3_^−^ produced by enhanced AO in the euphotic zone would directly contribute to refuel NO_3_^−^ up-taken by phytoplankton [41]. At upwelling-influenced Stn. D102, the new produced recycled NO_x_^−^ at three different depths (5, 25 and 50 m, Table 2) were 11%, 15%, and 18%, respectively, which were estimated with an AO rate at 1 day and 1 L and compared with the ambient inorganic nitrogen concentrations (NH_4_^+^ + NO_x_^−^). However, at non-upwelling Stn. D104, those proportions were reduced to only 0.9%, 1.5%, and 0.5%, respectively, indicating that NH_4_^+^ oxidation was important to maintain the high primary productions at upwelling-influenced stations [21].

Ammonia oxidation rates increased with the water depths observed in our study, and they had a positive correlation (R = 0.7827, *p* < 0.05) with the water depths in the upwelling stations. This process was previously observed and was attributed to light inhibition [42] and competition with phytoplankton for NH_4_^+^ in the photic zone [38]. However, the source of ambient NH_4_^+^ derived from the microbial decomposition of high dissolved organic matter should not be ignored. Dissolved organic matter might be highly remineralized by the increased phytoplankton biomass which occurred during upwelling [43], and the decomposition-released inorganic NH_4_^+^ would subsequently result in rapid ammonia oxidation mediated by AOA due to their high ammonia affinity [41]. Ammonia oxidation rates, which increased with water depth, were also found in the northern South China Sea where the Kuroshio Current frequently intrudes [42] and the Peruvian coastal upwelling regions [44] as well.

AOA are the key players in the marine nitrogen cycle; however, not all AOA clusters were actively involved in ammonia oxidization [38,45]. The *amoA* gene abundance of clusters WCA and WCB had no correlation with the nitrification rates throughout the water column in the Pacific Ocean [35]. Despite limited data with four stations in our study, AO rates had a significant positive correlation (R = 0.9998, *p* < 0.01) with the proportion of WCA (including WCA I, II, III, Ⅳ, and Ⅶ) at the cDNA level at ~25 m depths in the upwelling region; however, no significant correlation was found at the DNA level. This further proved that the actually active AOA sublineages might be concealed on DNA-based studies [35,36], and suggested that it was the WCA cluster rather than SCM1-like which contributed to the in situ ammonia oxidization, although the latter might have had a prompt response to the coastal upwelling, as demonstrated by its increased proportions at the cDNA level at upwelling stations.

The information above suggested that the interaction between different AOA clusters/sublineages and varied environmental parameters resulted from coastal upwelling could be very complex. We found that coastal upwelling could significantly increase the *amoA* gene abundances and induce the population shifts of the AOA community, accompanied by higher AO rates in the deeper water. Our study highlighted that coastal upwelling, as an important physical force, should be considered in future studies to achieve a comprehensive estimation of the global nitrogen budget and cycling.

## Figures and Tables

**Table 1:** Sequencing information and diversity parameters of the AOA *amoA* gene in the eastern Hainan Island during summer.

Samples	No. of Seqs *	No. of OTUs	*J* (Pielous’ Evenness)	Shannon	Simpson	Coverage (%)
D001—5m	1200	47	0.41	1.59	0.62	97.82
cD001—5m	1200	50	0.49	1.91	0.78	97.92
D001—25m	1200	68	0.42	1.79	0.67	96.81
cD001—25m	1200	30	0.49	1.68	0.74	98.81
DD101—5m	1200	43	0.33	1.23	0.51	98.18
cDD101—5m	1200	56	0.58	2.33	0.84	98.07
DD101—25m	1200	38	0.44	1.60	0.68	98.38
cDD101—25m	1200	61	0.42	1.73	0.67	97.29
D102—25m	1200	85	0.52	2.31	0.79	96.08
cD102—25m	1200	65	0.46	1.90	0.74	96.91
D104—25m	1200	138	0.83	4.09	0.97	97.64
cD104—25m	1200	54	0.53	2.12	0.79	97.78

c: cDNA; *: normalized quality reads.

**Table 2:** The vertical ambient NH_4_^+^, NO_x_^−^ concentrations and ammonia-oxidizing rates in upwelling and non-upwelling regions in the eastern Hainan Island during summer. The errors represent standard deviations.

	Station	Depth (m)	Ambient NH_4_^+^ (nM)	Ambient NO_x_^−^ (μM)	AO rate (nmol L^−1^ d^−1^)
Upwelling	D001	25	72.75 ± 2.58	0.20 ± 0.08	2.64 ± 0.29
		75	97.13 ± 8.40	7.01 ± 1.61	112.00 ± 14.27
	D102	5	55.93 ± 10.33	0.13 ± 0.08	1.53 ± 1.11
		25	57.56 ± 5.22	0.48 ± 0.40	5.63 ± 5.84
		50	86.57 ± 6.84	0.87 ± 0.26	11.97 ± 5.37
	DD101	25	19.98 ± 0.68	0.23 ± 0.12	0.77 ± 0.47
		75	51.28 ± 3.99	3.93 ± 2.61	52.00 ± 13.25
Non-Upwelling		5	13.62 ± 0.69	0.19 ± 0.03	0.13 ± 0.01
	D104	25	14.26 ± 0.29	3.83 ± 1.54	4.11 ± 5.93
		50	9.98 ± 0.15	8.51 ± 2.41	3.31 ± 1.67

## Data Availability

All the *amoA* sequences obtained from this study were deposited in the National Center for Biotechnology Information (NCBI) Sequence Read Archive (SRA) under the accession number PRJNA732652.

## Supplementary Materials

The following are available online at https://www.mdpi.com/article/10.3390/microorganisms10050952/s1, Table S1: Physical-chemical parameters of the sampling stations in the EHU during summer.

## References

[1] Dang H., Zhou H., Yang J., Ge H., Jiao N., Luan X., Zhang C., Klotz M.G. (2013). Thaumarchaeotal Signature Gene Distribution in Sediments of the Northern South China Sea: An Indicator of the Metabolic Intersection of the Marine Carbon, Nitrogen, and Phosphorus Cycles?. Appl. Environ. Microbiol..

[2] Mincer T.J., Church M.J., Taylor L.T., Preston C., Karl D.M., DeLong E.F. (2007). Quantitative distribution of presumptive archaeal and bacterial nitrifiers in Monterey Bay and the North Pacific Subtropical Gyre. Environ. Microbiol..

[3] Santoro A.E., Casciotti K., Francis C. (2010). Activity, abundance and diversity of nitrifying archaea and bacteria in the central California Current. Environ. Microbiol..

[4] Beman J.M., Sachdeva R., Fuhrman J. (2010). Population ecology of nitrifying Archaea and Bacteria in the Southern California Bight. Environ. Microbiol..

[5] Newell S., Fawcett S., Ward B.B. (2013). Depth distribution of ammonia oxidation rates and ammonia-oxidizer community composition in the Sargasso Sea. Limnol. Oceanogr..

[6] Francis C.A., Roberts K.J., Beman J.M., Santoro A.E., Oakley B.B. (2005). Ubiquity and diversity of ammonia-oxidizing archaea in water columns and sediments of the ocean. Proc. Natl. Acad. Sci. USA.

[7] Beman J.M., Popp B., Francis C. (2008). Molecular and biogeochemical evidence for ammonia oxidation by marine Crenarchaeota in the Gulf of California. ISME J..

[8] Santoro A.E., Saito M.A., Goepfert T.J., Lamborg C., Dupont C.L., DiTullio G.R. (2017). Thaumarchaeal ecotype distributions across the equatorial Pacific Ocean and their potential roles in nitrification and sinking flux attenuation. Limnol. Oceanogr..

[9] Cheung S., Mak W., Xia X., Lu Y., Cheung Y., Liu H. (2019). Overlooked Genetic Diversity of Ammonia Oxidizing Archaea Lineages in the Global Oceans. J. Geophys. Res. Biogeosci..

[10] Könneke M., Bernhard A.E., José R., Walker C.B., Waterbury J.B., Stahl D.A. (2005). Isolation of an autotrophic ammonia-oxidizing marine archaeon. Nature.

[11] Biller S.J., Mosier A.C., Wells G.F., Francis C.A. (2012). Global Biodiversity of Aquatic Ammonia-Oxidizing Archaea is Partitioned by Habitat. Front. Microbiol..

[12] Santoro A.E., Richter R.A., Dupont C.L. (2019). Planktonic Marine Archaea. Annu. Rev. Mar. Sci..

[13] Pester M., Rattei T., Flechl S., Grngrft A., Wagner M. (2012). Amoa-based consensus phylogeny of ammonia-oxidizing archaea and deep sequencing of amoA genes from soils of four different geographic regions. Environ. Microbiol..

[14] Sintes E., Bergauer K., De Corte D., Yokokawa T., Herndl G.J. (2013). Archaeal amoA gene diversity points to distinct biogeography of ammonia-oxidizing Crenarchaeota in the ocean. Environ. Microbiol..

[15] Molina V., Belmar L., Levipan H.A., Ramírez-Flandes S., Anguita C., Galán A., Montes I., Ulloa O. (2020). Spatiotemporal Distribution of Key Pelagic Microbes in a Seasonal Oxygen-Deficient Coastal Upwelling System of the Eastern South Pacific Ocean. Front. Mar. Sci..

[16] Lu Y., Xia X., Cheung S., Jing H., Liu H. (2019). Differential Distribution and Determinants of Ammonia Oxidizing Archaea Sublineages in the Oxygen Minimum Zone off Costa Rica. Microorganisms.

[17] Happel E., Bartl I., Voss M., Riemann L. (2018). Extensive nitrification and active ammonia oxidizers in two contrasting coastal systems of the Baltic Sea. Environ. Microbiol..

[18] Ward B., Zafiriou O. (1988). Nitrification and nitric oxide in the oxygen minimum of the eastern tropical North Pacific. Deep Sea Res. Part A Oceanogr. Res. Pap..

[19] Dolmatova L.S., Eliseykina M.G., Timchenko N.F., Kovaleva A.L., Shitkova O.A. (2003). Generation of reactive oxygen species in different fractions of the coelomocytes of holothurianEupentacta fraudatrix in response to the thermostable toxin ofYersinia pseudotuberculosis in vitro. Chin. J. Oceanol. Limnol..

[20] Gan J., Cheung A., Guo X., Li L. (2009). Intensified upwelling over a widened shelf in the northeastern South China Sea. J. Geophys. Res. Earth Surf..

[21] Liu S., Hong B., Wang G., Wang W., Xie Q., Ni Z., Yu L., Jiang H., Long T., Xu H. (2020). Physical structure and phytoplankton community off the eastern Hainan coast during summer 2015. Acta Oceanol. Sin..

[22] Schloss P.D., Westcott S.L., Ryabin T., Hall J.R., Hartmann M., Hollister E.B., Lesniewski R.A., Oakley B.B., Parks D.H., Robinson C.J. (2009). Introducing mothur: Open-Source, Platform-Independent, Community-Supported Software for Describing and Comparing Microbial Communities. Appl. Environ. Microbiol..

[23] Santoro A.E., Dupont C.L., Richter R.A., Craig M.T., Carini P., McIlvin M.R., Yang Y., Orsi W.D., Moran D.M., Saito M.A. (2015). Genomic and proteomic characterization of “*Candidatus Nitrosopelagicus brevis*: An ammonia-oxidizing archaeon from the open ocean. Proc. Natl. Acad. Sci. USA.

[24] Oksanen J., Blanchet F.G., Kindt M.R., Legendre P., McGlinn D., Minchin P.R., O’Hara R.B., Simpson G.L., Solymos P.M., Stevens H.H. (2019). Vegan: Community Ecology Package. https://CRAN.R-project.org/package.

[25] Logares R., Audic S., Bass D., Bittner L., Boutte C., Christen R., Claverie J.-M., Decelle J., Dolan J.R., Dunthorn M. (2014). Patterns of Rare and Abundant Marine Microbial Eukaryotes. Curr. Biol..

[26] Jing H., Cheung S.Y., Xia X., Suzuki K., Nishioka J., Liu H. (2017). Geographic Distribution of Ammonia-Oxidizing Archaea along the Kuril Islands in the Western Subarctic Pacific. Front. Microbiol..

[27] Tamura K., Stecher G., Peterson D., Filipski A., Kumar S. (2013). MEGA6: Molecular Evolutionary Genetics Analysis Version 6.0. Mol. Biol. Evol..

[28] Ward B.B., Kilpatrick K.A., Renger E.H., Eppley R.W. (1989). Biological nitrogen cycling in the nitracline. Limnol. Oceanogr..

[29] Sigman D.M., Casciotti K.L., Andreani M., Barford C., Galanter M., Böhlke J.K. (2001). A Bacterial Method for the Nitrogen Isotopic Analysis of Nitrate in Seawater and Freshwater. Anal. Chem..

[30] Casciotti K.L., Sigman D.M., Hastings M.G., Böhlke J.K., Hilkert A. (2002). Measurement of the Oxygen Isotopic Composition of Nitrate in Seawater and Freshwater Using the Denitrifier Method. Anal. Chem..

[31] Knapp A.N., Sigman D.M., Lipschultz F. (2005). N isotopic composition of dissolved organic nitrogen and nitrate at the Bermuda Atlantic Time-series Study site. Glob. Biogeochem. Cycles.

[32] Wickham H. (2016). Ggplot2: Elegant Graphics for Data Analysis.

[33] Agogué H., Brink M., Dinasquet J., Herndl G.J. (2009). Erratum: Major gradients in putatively nitrifying and non-nitrifying Archaea in the deep North Atlantic. Nature.

[34] Tolar B.B., King G.M., Hollibaugh J.T. (2013). An Analysis of Thaumarchaeota Populations from the Northern Gulf of Mexico. Front. Microbiol..

[35] Smith J.M., Damashek J., Chavez F.P., Francis C.A. (2016). Factors influencing nitrification rates and the abundance and transcriptional activity of ammonia-oxidizing microorganisms in the dark northeast Pacific Ocean. Limnol. Oceanogr..

[36] Lu Y., Cheung S.Y., Chen L., Kao S.-J., Xia X., Gan J., Dai M., Liu H. (2020). New insight to niche partitioning and ecological function of ammonia oxidizing archaea in subtropical estuarine ecosystem. Biogeosciences.

[37] Merbt S.N., Stahl D.A., Casamayor E.O., Martí E., Nicol G.W., Prosser J.I. (2011). Differential photoinhibition of bacterial and archaeal ammonia oxidation. FEMS Microbiol. Lett..

[38] Smith J.M., Chavez F.P., Francis C. (2014). Ammonium Uptake by Phytoplankton Regulates Nitrification in the Sunlit Ocean. PLoS ONE.

[39] Qin W., Amin S.A., Martens-Habbena W., Walker C.B., Urakawa H., Devol A.H., Ingalls A.E., Moffett J.W., Armbrust E.V., Stahl D.A. (2014). Marine ammonia-oxidizing archaeal isolates display obligate mixotrophy and wide ecotypic variation. Proc. Natl. Acad. Sci. USA.

[40] Martens-Habbena W., Berube P.M., Urakawa H., de la Torre J.R., Stahl D.A. (2009). Ammonia oxidation kinetics determine niche separation of nitrifying Archaea and Bacteria. Nature.

[41] Xu M.N., Zhang W., Zhu Y., Liu L., Zheng Z., Wan X.S., Qian W., Dai M., Gan J., Hutchins D.A. (2018). Enhanced Ammonia Oxidation Caused by Lateral Kuroshio Intrusion in the Boundary Zone of the Northern South China Sea. Geophys. Res. Lett..

[42] Ward B.B., Capone D.G., Bronk D.A., Mulholland M.R., Carpenter E.J. (2008). Nitrification in the marine environment. Nitrogen in the Marine Environment.

[43] Wetz M., Wheeler P. (2004). Response of bacteria to simulated upwelling phytoplankton blooms. Mar. Ecol. Prog. Ser..

[44] Fernández C., Farias L., Alcaman M. (2009). Primary production and nitrogen regeneration processes in surface waters of the Peruvian upwelling system. Prog. Oceanogr..

[45] Church M.J., Wai B., Karl D.M., DeLong E.F. (2010). Abundances of crenarchaeal amoA genes and transcripts in the Pacific Ocean. Environ. Microbiol..

