# Supplementary material for: Distribution and Oxidation Rates of Ammonia-Oxidizing Archaea Influenced by the Coastal Upwelling off Eastern Hainan Island
Source: Microorganisms. 2022 Apr 30;10(5):952. doi: 10.3390/microorganisms10050952 (PMC9143208; doi:10.3390/microorganisms10050952)
Supplement: Supplementary file 1 [file microorganisms-10-00952-s001.zip › microorganisms-1670211-supplementary.pdf]

microorganisms

MDPI

Supplementary material

Table S1. Physic-chemical parameters of the sampling stations in the EHU in summer.

Station	longitude (°E)	latitude (°N)	Depths (m)	Temp. (°C)	Sal. (‰)	NH_4^+ (nmol L^{-1})	NO_2^- (µmol L^{-1})	NO_x^- (µmol L^{-1})	PO_4^{3-} (µmol L^{-1})	SiO_3^{2-} (µmol L^{-1})
**D001**	**110.717**	18.974	5	26.82	33.50	27.24	BLQ	BLQ	BLQ	2.86
			25	22.18	34.27	45.59	0.04	0.15	BLQ	3.62
**D102**	110.852	19.280	25	24.22	34.03	BLQ	0.13	0.35	0.09	3.46
**DD101**	110.238	18.360	5	26.93	33.56	BLQ	BLQ	BLQ	BLQ	2.43
			25	23.87	34.01	BLQ	BLQ	BLQ	BLQ	2.71
**D104**	111.672	18.731	25	28.99	33.38	BLQ	BLQ	BLQ	BLQ	1.81

NO_x^-: NO_2^- + NO_3^-.

BLQ: below the limit of quantitation.
